# Socio-demographic, clinical and health behavior correlates of sitting time in older adults

**DOI:** 10.1186/s12889-015-1426-x

**Published:** 2015-01-31

**Authors:** Joilson Meneguci, Jeffer Eidi Sasaki, Álvaro da Silva Santos, Lucia Marina Scatena, Renata Damião

**Affiliations:** Department of Sport Sciences, Federal University of the Triângulo Mineiro, Av. Getúlio Guaritá, 159, Bairro Nossa Senhora da Abadia, Uberaba, MG CEP: 38025-440 Brazil; Graduate Program in Physical Education, Federal University of Triângulo Mineiro, Uberaba, Minas Gerais Brazil; Department of Nursing, Graduate Program in Healthcare, Federal University of Triângulo Mineiro, Uberaba, Minas Gerais Brazil; Department of Social Medicine, Graduate Program in Technological Innovation, Federal University of Triângulo Mineiro, Uberaba, Minas Gerais Brazil; Department of Nutrition, Graduate Program in Physical Education, Federal University of Triângulo Mineiro, Uberaba, Minas Gerais Brazil

**Keywords:** Sedentary behavior, Sitting time, Older adults, Epidemiology

## Abstract

**Background:**

Identifying correlates of sedentary behavior in older adults is of major importance to healthcare. To our knowledge, there are no population studies in Latin America examining which factors are associated to high sitting time in older adults. Thus, the purpose of this study is to identify socio-demographic, clinical, and health behavior correlates of sitting time in a representative sample of older adults living in Southeastern Brazil.

**Methods:**

A cross-sectional study was conducted in twenty-four municipalities of the Triangulo Mineiro region in the State of Minas Gerais, Southeastern Brazil. A structured questionnaire was applied to obtain information on socio-demographic, clinical, and health behavior factors. Overall sitting time was assessed using a self-report instrument. A Multiple Correspondence Analysis was used to verify the association of sitting time with socio-demographic, clinical, and health behavior factors.

**Results:**

3,296 older adults (61.5% women and 38.5% men) were included in the analysis. The overall median was 240.0 minutes of sitting time/day. The Multiple Correspondence Analysis showed that the group with the highest sitting time presented the following characteristics: women, age greater than 70 years, unschooled status, arterial hypertension, diabetes mellitus, use of medication, poor self-rated health, dependence in basic activities of daily living, and absence of regular physical activity.

**Conclusion:**

This study reveals that socio-demographic, clinical, and health behavior factors are associated with high sitting time in older adults from Southeastern Brazil. The results may help to identify older adults that should be targeted in interventions aiming at reducing sitting time.

## Background

Life expectancy has increased substantially in developed countries in the last fifty years. This same phenomenon is taking place in developing countries and the impact of this demographic transition is one of the major challenges in the field of Public Healthcare [1,2].

In addition to the demographic transition, technological advances have facilitated performance of everyday tasks but at the same time are leading to a less active lifestyle [3]. This can be clearly seen with transportation habits in developed countries. For example, Sugiyama et al. [4] observed that 66% of transportation trips in Sidney, Australia, are made by car, with 14% of these trips lasting for two or more hours. Developing countries have been experiencing an increase in access to automobiles and will likely demonstrate similar trends in transportation habits in the near future. Along with greater car utilization, many other technological advances have become more accessible to the Brazilian population and may result in increases in sedentary behavior.

Studies have shown that prolonged sedentary behavior, independent of physical activity level, is associated with various negative health conditions such as cardiovascular disease [5], obesity [6], diabetes mellitus [7], cancer [8], metabolic syndrome [9], and psychological distress [10].

Self-reported sitting time has been used in many studies as a specific indicator of sedentary behavior [10,11]. In older adults, recent research conducted in Australia [12] and Spain [13] revealed an association between self-reported sitting time and all-cause mortality. Moreover, prolonged sitting time has been associated with metabolic syndrome [14], excess weight [15], reductions in physical, social, psychological factors [16] and quality of life [17] in older adults.

From this evidence, it is essential to identify correlates of sitting time in older adults so that future interventions can target those individuals at higher likelihood for engaging in sedentary behaviors [18]. Although some research has traced this profile in older adults [18-25], there is still a need for further investigations in different countries and regions from the globe To our knowledge, there are no population studies in Latin America examining correlates of sitting time in older adults. Thus, the purpose of this study is to identify socio-demographic, clinical, and health behavior factors that are associated with high sitting time in a representative sample of older adults living in Southeastern Brazil.

## Methods

### Study population

This cross-sectional study is part of the research project entitled “Health profile of the elderly population of the municipalities of the Regional Healthcare Administration - Uberaba/Minas Gerais”. The project contemplated a population of 79,924 people aged 60+ years [26] living in municipalities located in the Triângulo Mineiro region, in the State of Minas Gerais, Southeastern Brazil. This study covered 24 municipalities, representing a territorial area of 33,594.041 Km^2^ with an average human development index of 0.717 [27].

### Participants

Calculation of the representative sample took as reference the population of older adults of each municipality [26] and the following parameters: sampling error of 0.05, confidence interval of 95% and the proportion of the population in each municipality (population of older adults divided by the total population). The calculation indicated that a minimum of 3,198 older adults was necessary for reaching representativeness of the population of interest. A simple random sampling process was used to recruit the participants.

Inclusion criteria for this study were: age ≥ 60 years; agreement to participate in the study by signing an informed consent form; achievement of the minimum score on the Mini-Mental State Examination [28,29] and ability to walk without the use of assistive devices or using the help of a cane or walker. Exclusion criteria were: being dependent on a wheelchair, severe hearing or sight disability that considerably hindered communication, and being temporarily or permanently bedridden.

### Data collection

Data were collected from May 2012 to April 2013. Participants answered a structured questionnaire applied by trained interviewers containing questions on socio-demographic information, clinical factors, health behaviors, and sitting time. Body mass (kilograms) and height (meters) were measured on the same day of the interview.

The socio-demographic factors included age, sex (male, female), monthly household income (< $308.18, ≥ $308.18 and ≤ $924.54, > $924.54), living arrangements (living alone, living with others), and schooling (schooled, unschooled). Given the low-educational level of our sample, we decided to adopt a simple classification of the participants as unschooled if they have never been to school, and schooled if they ever attended school.

Clinical factors included: presence of arterial hypertension and/or diabetes mellitus, use of medication (yes, no), falls in the last year (yes, no), self-rated health, ability to perform basic activities of daily living, and body mass index (BMI).

Self-rated health was assessed using a scale that is commonly employed in epidemiological studies assessing sedentary behavior in older adults [12,16,25]. Self-rated health was first evaluated using a four-category scale (excellent, good, fair, poor) and was later reclassified into two categories: good (excellent and good) and poor (fair and poor). Ability to perform basic activities of daily living was assessed using the Brazilian version of the ‘Index of Independence in Activities of Daily Living’ (Index of ADL) [30,31]. The ‘Index of ADL’ questionnaire includes questions on the following activities of daily living (ADL): bathing, dressing, toileting, transfer, continence and feeding. In this study, participants were classified as independent if they did not need any assistance in performing any of ADL tasks, and dependent if they needed assistance in one or more ADLs. Based on the body mass index, participants were categorized as underweight (BMI <18.5 kg/m2), normal weight (BMI 18.5-24.9 kg/m2), overweight (BMI 25.0-29.9 kg/m2) or obese (BMI ≥ 30.0 kg/m^2^) [32].

Health behaviors were assessed with questions pertaining to smoking, alcohol consumption and regular physical activity. The questions were: *1) Do you currently smoke?, 2) Do you currently consume alcoholic beverages/drinks?, and 3) Do you engage in physical activity regularly?* Answers to these questions were either yes or no.

Overall sitting time was evaluated using questions regarding time spent seated on a usual weekday and a usual weekend day according to the International Physical Activity Questionnaire [33] validated for the Brazilian elderly population [34,35].

### Data analysis

Data were entered in duplicate on Excel software, version 2007, and the statistical analyses were performed using the STATISTICA software (StatSoft, version 10.0).

Descriptive analysis was performed for all variables. The overall sitting time in minutes/day was determined from the weighted average of the time spent seated on a weekday and on a weekend day. Participants were divided into two groups: the first group (G1) corresponded to values under the 75th percentile and the second group (G2) corresponded to values of the 75th percentile or above. This approach was used because previous research suggests that individuals at the highest quartile of sitting time are those with the highest risks for adverse health outcomes [14,17].

A Multiple Correspondence Analysis was used for examining the association of sitting time with socio-demographic, clinical, and health behavior factors. The Multiple Correspondence Analysis method projects data into space-dimensions and searches for patterns in the dataset, helping to identify the variables more closely associated with different groups. A matrix of eigenvalues was determined to identify a combination of variables that presented more stability in the factorial plan and explained the largest percentage of variability in the dataset. This was verified by the square of the cosine (Cos^2^) of the angle between the variables and respective dimensions. The candidate variables in the factorial plan for the bivariate analysis of sitting time with socio-demographic, clinical and health behavior factors were determined by statistical significance (p < 0.05). The criterion adopted for variables to remain in the factorial plan was the selection of variables with greater discrimination in each dimension. The number of dimensions was chosen by analyzing the decline of eigenvalues [36].

We chose the Multiple Correspondence Analysis because of its statistical robustness and clear visual interpretation of the graphs, which helps to confirm associations or similarities between variables. The Multiple Correspondence Analysis is suitable for population-based studies as suggested by Carvalho [36].

### Ethics statement

Volunteers were informed about the objectives and procedures of the study and provided written informed consent. The study protocol and procedures are in accordance with the Declaration of Helsinki and were previously approved by the Human Research Ethics Committee of the Federal University of Triângulo Mineiro (Ruling no. 1640/2010).

## Results

The initial sample was comprised of 3,430 older adults aged 60+ years. A total of 134 were excluded from the study due to incomplete data, resulting in a final sample of 3,296 older adults with a mean age of 70 ± 7.29 years (age range: 60 to 96 years). The overall median for sitting time (25th, 75th percentile) was 240.00 (137.14, 330.00) minutes/day.

According to Table 1, older adults from this study were predominantly women (61.5%; n = 2,026) between 60 and 69 years of age (52.7%; n = 1,735), had some schooling (71.1%; n = 2,343), had a monthly household income between $308.18 and $924.54 (71.0%; n = 2,339), and lived with others (91.8%, n = 3,027).

**Table 1:** **Socio-demographic characteristics of those evaluated, grouped according to sitting time**

	**Total (n = 3,296)**		**G1 (n = 2,464)**		**G2 (n = 832)**		
**Variables**	**n**	**%**	**n**	**%**	**n**	**%**	p-value*
**Sex**							0.036
Male	1,270	38.5	924	37.5	346	41.6	
Female	2,026	61.5	1,540	62.5	486	58.4	
**Age**							0.000
60-69 years	1,735	52.7	1,345	54.6	390	46.9	
70-79 years	1,174	35.6	863	35.0	311	37.4	
≥80 years	387	11.7	256	10.4	131	15.7	
**Schooling**							0.009
Schooled	2,343	71.1	1,781	72.3	562	67.5	
Unschooled	953	28.9	683	27.7	270	32.5	
**Monthly household income**							0.394
< $308.18	568	17.2	416	16.9	152	18.3	
≥ $308.18 and ≤ $924.54	2,339	71.0	1,764	71.6	575	69.1	
> $924.54	389	11.8	284	11.5	105	12.6	
**Living arrangements**							0.139
Living alone	269	8.2	191	7.8	78	9.4	
Living with other	3,027	91.8	2,273	92.2	754	90.6	

*Chi-square.

G1: Sitting time < 330 minutes/day.

G2: Sitting time ≥ 330 minutes/day.

In terms of clinical factors, 65.9% (n = 2,171) suffered from arterial hypertension, 19.9% (n = 655) had diabetes mellitus, 87.3% (n = 2,877) used medication, 52.1% (n = 1,716) had poor self-rated health, 27.5% (n = 905) had a fall in the previous year, 14.6% (n = 481) were dependent in basic activities of daily living, and 25.0% (n = 826) were obese (Table 2).

**Table 2:** **Clinical characteristics of older adults grouped according to sitting time**

	**Total (n = 3,296)**		**G1 (n = 2,464)**		**G2 (n = 832)**		
**Variables**	**n**	**%**	**N**	**%**	**n**	**%**	p-value*
**Arterial hypertension**							0.001
Yes	2,171	65.9	1,583	64.2	588	70.7	
No	1,125	34.1	881	35.8	244	29.3	
**Diabetes mellitus**							0.002
Yes	655	19.9	459	18.6	196	23.6	
No	2,641	80.1	2,005	81.4	636	76.4	
**Use of medication**							0.044
Yes	2,877	87.3	2,134	86.6	743	89.3	
No	419	12.7	330	13.4	89	10.7	
**Falls**							0.162
Yes	905	27.5	661	26.8	244	29.3	
No	2,391	72.5	1,803	73.2	588	70.7	
**Self-rated health**							0.002
Good	1,580	47.9	1,219	49.5	361	43.4	
Poor	1,716	52.1	1,245	50.5	471	56.6	
**Basic activities of daily living**							0.000
Dependent	481	14.6	309	12.5	172	20.7	
Independent	2,815	85.4	2,155	87.5	660	79.3	
**Body mass index**							0.011
Underweight	104	3.2	70	2.9	34	4.1	
Normal weight	1,121	34.0	848	34.4	273	32.8	
Overweight	1,245	37.8	956	38.8	289	34.7	
Obese	826	25.0	590	23.9	236	28.4	

*Chi-square.

G1: Sitting time < 330 minutes/day.

G2: Sitting time ≥ 330 minutes/day.

As for health behaviors, 15.2% (n = 503) were smokers, 15.8% (n = 522) reported consuming alcoholic beverages, and 43.1% (n = 1,432) did not engage in regular physical activity (Table 3).

**Table 3:** **Health behaviors of older adults grouped according to sitting time**

	**Total (n = 3,296)**		**G1 (n = 2,464)**		**G2 (n = 832)**		
**Variables**	**n**	**%**	**N**	**%**	**n**	**%**	p-value*
**Smoking**							0.518
Yes	500	15.2	368	14.9	132	15.9	
No	2,796	84.8	2,096	85.1	700	84.1	
**Alcohol consumption**							0.933
Yes	522	15.8	391	15.9	131	15.7	
No	2,774	84.2	2,073	84.1	701	84.3	
**Regular physical activity**							0.001
Yes	1,875	56.9	1,474	59.8	401	48.2	
No	1,421	43.1	990	40.2	431	51.8	

*Chi-square.

G1: Sitting time < 330 minutes/day.

G2: Sitting time ≥ 330 minutes/day.

According to Tables 1, 2, and 3, the variables sex, age, schooling, arterial hypertension, diabetes mellitus, use of medication, self-rated health, basic activities of daily living, BMI and regular physical activity presented significance (*p* < 0.05) for associations with sitting time and were candidates for the Multiple Correspondence Analysis.

In the analysis of the factorial plan, BMI was not among the combination of variables that showed greater stability and explanatory power for the percentage of variability in sitting time. Therefore, BMI was disregarded from further analyses. Variables with greater discrimination in each dimension and which therefore remained in the factorial plan were: sex, age, schooling, arterial hypertension, diabetes mellitus, use of medication, self-rated health, basic activities of daily living, and regular physical activity.

The analysis of the eigenvalues favored dimensions 1 and 2 with values of 0.445 and 0.379, respectively. Table 4 presents the results of the Multiple Correspondence Analysis with the variables and their respective values of absolute contribution (Cos^2^) to dimensions 1 and 2. Dimension 1 consisted of clinical factors with the highest contribution to high sitting time (G2). These clinical factors were: arterial hypertension, diabetes mellitus, use of medication, self-rated health, and basic activities of daily living. Dimension 2 consisted of socio-demographic factors with the highest contribution to high sitting time (G2). These socio-demographic factors were: age and schooling.

**Table 4:** **Absolute contribution of the variables to total sitting time according to correspondence analysis in the factorial plan**

	**Cos** ^**2**^	**Cos** ^**2**^	
**Variables**	**Dimension 1**	**Dimension 2**	**Dimension**
**Sex**			1
Male	0.178487	0.038911	
Female	0.178487	0.038911	
**Age**			2
60-69 years	0.005844	0.295584	
70-79 years	0.002907	0.045769	
≥80 years	0.001472	0.275643	
**Schooling**			2
Schooled	0.007240	0.367995	
Unschooled	0.007240	0.367995	
**Arterial hypertension**			1
Yes	0.501896	0.018283	
No	0.501896	0.018283	
**Diabetes mellitus**			1
Yes	0.200546	0.038845	
No	0.200546	0.038845	
**Use of medication**			1
Yes	0.530507	0.030613	
No	0.530507	0.030613	
**Self-rated health**			1
Good	0.215100	0.054552	
Poor	0.215100	0.054552	
**Basic activities of daily living**			1
Dependent	0.125627	0.107130	
Independent	0.125627	0.107130	
**Regular physical activity**			2
Yes	0.024448	0.223931	
No	0.024448	0.223931	

Squared cosine values may be interpreted as the correlation between the variable and the respective dimension.

**Figure 1:**
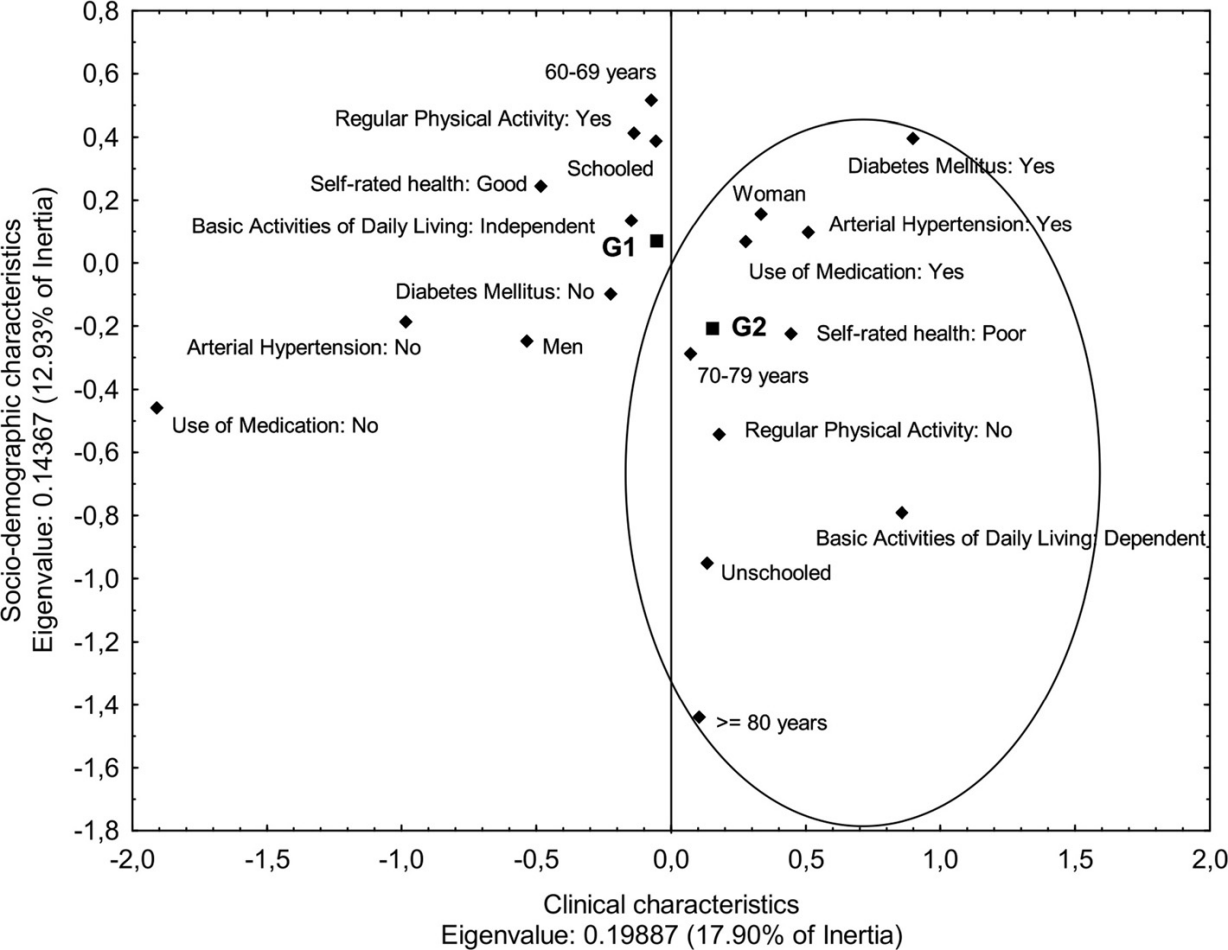
**Factorial plan of association of the socio-demographic, clinical, and health behaviors profile with sitting time.** Multiple Correspondence Analysis. G1: Sitting time < 330 minutes/day; G2: Sitting time ≥ 330 minutes/day.

The variables of dimensions 1 and 2 were found by using coordinates (X, Y) in the factorial plan, and explained 17.90% and 12.93% of the variability of the data, respectively (Figure 1). The proximity of the variables to G1 or G2 indicates the characteristics that each group is more likely to present. When analyzing the factorial plan, the following characteristics were observed for the group presenting 330 minutes or more of sitting time/day (G2): women between 70 and 79 years of age, poor self-rated health, medication use, high blood pressure, diabetes mellitus, absence of regular physical activity, dependence in basic activities of daily living, and unschooled status. The G2 group was closest to the Y axis and, consequently, more associated with dimension 2 which means that the categories of variables that made up this dimension (socio-demographic factors) were the variables that contributed the most to describe the G2 group. In contrast, the categories of variables not associated with G2 were associated with G1 (sitting time <330 minutes/day) as depicted in Figure 1.

## Discussion

To our knowledge, this is the first population-based study in a Latin American country examining the socio-demographic, clinical and health behavior correlates of sitting time in older adults. According to a recent systematic review, which aimed to identify research on sedentary behavior in individuals aged 60+ years, no study in Latin American countries was identified until 2013 [37]. Considering the fast population aging that is currently taking place in developing countries [1], it is essential to identify older adults at higher risks for developing adverse health conditions.

In this context, our study identified that 50% of the participants sat for approximately four hours every day. This result is somewhat similar to that by Dogra and Stathokostas [25] and Wallmann-Sperlich et al. [18], who found that half of the participants from their study spent four or more hours/day sitting. Conversely, in the study of Gómez-Cabello et al. [15], only 36.4% of men and 30.2% of women sat for four hours or more daily.

In our study, participants in the higher sitting time (≥330 minutes/day) group (G2) were in general: women over 70 years of age, unschooled, had arterial hypertension and/or diabetes, used medication, had poor self-rated health, were dependent in basic activities of daily living, and did not engage in regular physical activity.

These results are in line with previous research showing that sedentary behavior is influenced by several factors, including personal, social and cultural factors [38]. In Brazil, leisure time of older adults is one of the factors that may contribute to high sitting time. Brazilian older adults usually do not participate in the labor force [39], resulting in greater free time that can either be used to be physically active or to engage in sedentary behaviors.

Watching television is considered a leisure activity and is frequently reported as the most common sedentary activity in this age group [40,41]. Prolonged television time has been previously associated with place of residence (urban, suburban and regional) in older adults from Japan [23]. Given that our sample was comprised of participants living in urban areas, we believe that most of the self-reported sitting time may have been due to television viewing.

A factor that may influence sedentary behavior is the built environment [42,43]. A study demonstrated that prolonged television viewing was associated with perceived aspects of the neighborhood environment, including heavy traffic and crime, lack of neighborhood lighting, and poor scenery [44]. In addition, several studies demonstrate that the built environment also influences physical activity of older adults [45,46]. Therefore, it is likely that a non-favorable built environment may prevent older adults from replacing some of the sitting time with physical activity. Although we did not evaluate the influence of the built environment on sitting time of older adults in this study, we encourage future studies to do so, as such information is important for the successful implementation of future interventions and public health policies.

Sedentary behavior may also be influenced by gender but there is no consensus within the research community [47-49]. In the study by Chastin et al. [50], it was identified that the reasons leading older women to spend high amounts of time sitting are: physical complaints, lack of facilities and environmental stimuli, peer and societal pressure, pleasure and relaxation, and mental health reasons. Besides these factors, culturally in Brazil, older women were not taught to pursue activities outside home (e.g., shopping, banking, physical exercise). In addition, intervention programs offering physical exercise for older Brazilians are usually concentrated in the group aged 60-69 years [51,52]. Future research should identify the environmental and social factors that influence sedentary behavior in women aged 70+ years. This population should also receive attention in interventions aiming at reducing sitting time.Another socio-demographic factor that was associated with sitting time in this study was educational level. The unschooled status was associated with greater sitting time, suggesting that specific strategies to reduce sitting time in individuals without education may be necessary. These strategies may include the communication of the deleterious effects of sitting in a way that is simple and easy-to-comprehend. Appropriate tuning of health messages to the target population is important for allowing for correct understanding. In populations with low educational levels, the use of visual and graphic messages may be more effective than instructional texts.

Among the clinical factors associated with sitting time in the present study, it is possible to highlight some negative health outcomes, including arterial hypertension, diabetes mellitus, medication use, and poor self-rated health. The association of higher sitting time with arterial hypertension and diabetes mellitus corroborates the results of other cross-sectional studies that previously examined this subject [14,47,48,53]. Additionally, longitudinal studies carried out in Spain and the United States further supports that individuals spending more time in sedentary behavior have higher risks for hypertension and diabetes [7,54]. In the present study, we also found an association between sitting time and medication use. This is in line with the findings from the study by Silva et al. [55], who found that a higher number of steps/day were significantly associated with lower medication use in older women from Brazil. In relation to the association between sitting time and poor self-rated health, our results confirm the findings of previous studies conducted with older adults [25]. The study entitled ‘The 45 and Up Study’ examined the association of sitting time with self-rated health in 194,545 Australian older adults [56]. The results showed that individuals sitting <4 hours/day rated their health status as excellent [56].

In view of this evidence, interventions aiming at reducing time spent in sedentary behavior may be effective for achieving and maintaining good health by improving self-rated health and reducing medication use, hypertension and diabetes.

Sedentary behavior is also associated with reductions in components of physical fitness among the elderly population [49,57]. These reductions may increase the risk of falling [58] and functional disability [59]. In this study, we found no correlation between falls reported in the last year and sitting time. On the other hand, sitting time was significantly associated with dependence in activities of self-care: bathing, dressing, going to the bathroom, getting in and out of bed, eating, and control of the functions of urination or bowel movement, was associated with sitting time. These results suggest that interventions to reduce sedentary behavior may contribute to improvements of physical fitness components and, consequently, reduce the dependence in basic activities of daily living. According to Sardinha et al. [60], periodic and small interruptions to SB are likely to be of Importance in preventing the decline in physical function in older adults.

Finally, physical activity level tends to be lower with increasing age [61]. In this study, the regular practice of physical activity was negatively associated with sitting time, corroborating the findings of a previous study [17]. According to Buman et al. [61], there is a need to encourage older adults to perform light-intensity activity and reduce the amount of time spent in sedentary behaviors. Studies have shown that the combination of extensive time spent in sedentary behavior with physical inactivity potentiate health risks [48,56]. In this sense, when designing interventions for health promotion in older adults, both physical activity and sedentary behavior should be targeted, since they are independent behaviors.

In summary, this study identified the socio-demographic, clinical, and health behavior correlates of sitting time in a representative sample of older adults from Southeastern Brazil. The observed results are indicative of the need for future investigations to further identify other determinants of sedentary behavior in older adults from developing countries. In this context, longitudinal investigations may be of major importance. Given that sedentary behavior is considered a risk factor for chronic diseases and mortality, there is a need to develop interventions aiming at reducing this behavior. However, before intervening, it is always important to identify the ‘at-risk’ groups. Therefore, the present study contributes to identifying those older adults that should be prioritized in interventions in Southeastern Brazil aiming at reducing sitting time in this age group.

This study has strengths and limitations. The strengths of this study include the representative sample of older adults residents of twenty-four municipalities as well as the use of the Mini-Mental State Examination in order to ensure inclusion of only those older adults who could reliably answer the questions during the interview [27]. Another strength was that sitting time was assessed for both weekdays and weekend days. One of the limitations of this study was that we did not use an objective method to assess sedentary behavior (e.g. accelerometry). The use of a self-reported measure may have led to underestimation of the true sitting time in this study. Nevertheless, questionnaires are usually an appropriate methodology for large studies [62]. Another limitation was that no environmental assessment of the municipalities was made, which prevented us to make inferences about any associations of the built environment with time spent sitting in this study. Finally, the cross-sectional design of the study did not allow ascertaining a cause and effect relationship. It may be possible that reverse causality was responsible for the association between sitting time and health problems found in the present study.

## Conclusion

High sitting time in this study was associated with specific socio-demographic, clinical, and health behavior factors. The present study improves the understanding of the correlates of sitting time in older adults from a developing country. The results may be important for identifying the ‘at-risk’ groups of older adults when designing interventions strategies to reduce sitting time in developing countries with similar characteristics of Brazil.

## Abbreviations

BMI: Body mass index
ADL: Activities of daily living
